# Development of a patient centred, structured, individually tailored, multi-component intervention to promote rehabilitation and recovery after critical illness: content, theory, and construction.

**DOI:** 10.3310/nihropenres.14023.2

**Published:** 2025-10-06

**Authors:** Brenda O'Neill, Danny McAuley, Rachel Clarke, Sallyanne Duncan, Penelope Firshman, Ella Terblanche, Julie Bruce, Jill Costley, Bronwen Connolly, Judy Martina Bradley

**Affiliations:** 1 Institute of Nursing and Health Research, Ulster University, Belfast, Northern Ireland, UK; 2 Queen's University Belfast Wellcome-Wolfson Institute for Experimental Medicine, Belfast, Northern Ireland, UK; 3 University Hospitals Plymouth NHS Hospital,, Derriford Hospital, Plymouth, England, UK; 4 BHSCT Belfast, UK, Royal Victoria Hospital, Belfast, Northern Ireland, UK; 5 Surrey and Sussex Healthcare NHS Trust, Redhill, England, UK; 6 Principal Critical Care Dietitian, Health Sciences University, Bournemouth, UK; 7 University of Warwick, Warwick Clinical Trials Unit, Coventry, England, UK; 8 University Hospitals of Coventry and Warwickshire, Coventry, England, UK; 9 School of Health Sciences, The University of Melbourne, Melbourne, Australia

**Keywords:** Intervention Development, Rehabilitation, Critical illness, Intensive Care Unit (ICU), Recovery

## Abstract

**Background/aims:**

This paper describes the development (content, theory, and construction) of a patient-centered, structured, individually tailored, multicomponent intervention (the iRehab intervention) to promote rehabilitation and recovery after critical illness.

**Methods:**

The intervention was informed by the MRC framework for complex interventions and underpinned by existing literature and psychological theories. Key stakeholders included patients who had been in intensive care and multidisciplinary staff with experience in providing healthcare and undertaking research.

**Results:**

The final intervention includes four core components: 1. weekly discussion and guidance regarding symptom management; 2. targeted exercise and physical activity; 3. support for psychological well-being; 4. peer support and information provision. These are packaged as a program to support rehabilitation and recovery after ICU discharge.

Programme duration: Six weeks.

Format: Weekly one-to-one remote needs assessment to identify individual participant symptoms and provide management plans, exercises, and strategies to best support recovery. Participants are encouraged to attend weekly group-based remote exercise sessions and group-based remote support sessions (iRehab Café).

Mode of delivery: Remote delivery facilitated by online platforms such as Microsoft Teams or Zoom supported with video platform BEAM©, and delivery can also be supported by telephone. The preferred mode of remote delivery is agreed with the participant, and potential barriers to implementation are considered. Manuals are posted to all participants to support intervention delivery.

**Discussion/conclusion:**

This paper reports the content, theory and construction of the iRehab intervention. The iRehab intervention is currently being tested in a multicenter RCT (iRehab ISRCTN11266403), and the details reported in this paper will help with understanding of the intervention, interpretation of the findings, and replication of the intervention. Detailed intervention manuals will be available upon the completion of the trial.

## Plain Language Summary

**Background:**

People treated in intensive care units need a great deal of special care and support. After discharge from the hospital, some people find their muscles weak, and they cannot do everyday activities. They can also confuse memories of their time in the intensive care unit. Many patients are likely to experience PTSD-like symptoms.

Patients often report feeling abandoned after discharge because the intensive nature of their care has ended. This paper describes how a support and exercise rehabilitation program was developed. This is called the iRehab intervention.

**Methods:**

Many ex-ICU patients and their families helped us to find out what should be included in the iRehab intervention. We also included views from staff who provide care to patients in intensive care, as well as researchers and findings from other studies.

**Results:**

There are four main parts:

1. Discussion with the patient and iRehab sepcialist about how to manage symptoms or problems.

2. Help to do the right exercises and activities.

3. Support for emotional wellbeing.

4. Peer support and information provision e.g. sign-posting to relevant services, websites.

Each week, an iRehab specialist speaks to each patient to find out what their needs are. The iRehab specialist helps patients with treatments to help their recovery and rehabilitation. Patients get a manual with words and pictures to follow. This lasts for six weeks and can be delivered by a computer or telephone.

**Conclusion:**

This paper describes a collaborative approach for the development of the iRehab intervention. These details will help others, such as healthcare professionals, patients, and their families, to understand all parts of the intervention. The iRehab intervention is currently being tested in a large trial in the UK. When the trial has ended, the manuals that were created will be accessible to others.

## Background

People experience common and varied symptoms after critical care, often referred to as post intensive care syndrome (PICS) ^1^. There is a need to develop and evaluate interventions to support rehabilitation and recovery after discharge from the ICU, this has been ranked a key priority for research by patients, their families, and researchers ^1, 2^.

The National Institute of Clinical Excellence (NICE) guidelines recommend that the recovery pathway following critical illness should include regular assessment of physical and non-physical morbidity, goal setting, multiprofessional rehabilitation input according to individualized needs, and transition of information between care delivery stages ^3^. However, this practice has been applied poorly following hospital discharge, and rehabilitation services post critical illness in the UK are ad hoc, geographically inconsistent, and variable in terms of structure, content, and format of delivery ^4^. Feasible and alternative approaches to provide rehabilitation are needed to reach all those who could benefit and to optimize geographic access ^5^. More recently, technology-enabled care has been shown to be effective and accessible for the delivery of rehabilitation in other illnesses such as cardiac rehabilitation, balance rehabilitation in older people, chronic obstructive pulmonary disease, and in people after COVID ^6– 9^ but this needs to be tested in the context of rehabilitation after critical illness prior to widespread implementation in the NHS.

We developed the iRehab intervention which is currently being evaluated in a large-scale clinical trial with embedded process evaluation ^10^. The iRehab intervention is a patient-centered, structured, individually tailored, multicomponent intervention that aims to promote rehabilitation and recovery in the early period following hospital discharge after critical illness through enabling patient self-management, clarifying illness symptoms, offering targeted support and treatments, and co-developing action plans.

The aim of this paper is to describe the development of the iRehab intervention, including its theory, content, and construction. This information will help other clinicians and researchers to understand the rationale for the intervention, aid in the interpretation of trial findings, and allow replication of the intervention.

## Methods

### Patient and Public involvement

Patient and Public partners had a pivotal role from concept and throughout the design and development stages of this multicomponent intervention. This partnership continues while the intervention is tested during a RCT and its subsequent dissemination ^10^.

Patients and family members with lived experiences of critical illness indicated that they want access to a rehabilitation program that could support recovery after discharge home. They indicated that they would have attended such an intervention if it had been available to them, and this motivated them to be involved in the development of the iRehab intervention. Patient and Public partners inputted to qualitative research that also underpinned the intervention content. Based on their experiences and views, they identified that key facilitators of the intervention include supervision, tailoring of support and rehabilitation to personal needs, and access to manual/materials that can be referred to. They wanted the intervention to include help with motivation or mental health challenges if these were barriers to someone taking part, and help to modify exercises and activities depending on the stage of recovery. Other examples of active contributions by our Patient and Public partners were input into the content and design of the patent materials, the format for delivery of the intervention, and advice about practical issues such as access to computers. Patient and Public partners have formed our current iRehab Patient Advisory Group (PAG), which supports the implementation of this intervention in the iRehab trial ^10^. Together, we will plan dissemination to linked communities in formats that are accessible to a wide audience. To support the dissemination of this manuscript, two of our PAG members have co-written the plain language summary (see acknowledgements).

### Intervention development

This manuscript adheres to recommended guidance for explicit and comprehensive reporting as per the Template for Intervention Development and Replication (TiDIER) ^11^. The intervention embedded a range of influences to address the core elements ( *a-f*) recommended by the MRC for the development and testing of complex interventions ^12^. To ( *a*) ‘ *identify content*’ and ( *b*) ‘ *develop and refine the content, and programme theory’,* we included theories and evidence underpinning treatments, existing literature around post ICU outcomes, and patient feedback and experiences. Our key ( *c*) ‘ *stakeholders’* included multiprofessional staff with experience providing healthcare to post ICU patients and undertaking research, patient partners and a Patient Advisory Group (PAG) with former ICU survivors (including experience of mechanical ventilation), and relatives of ICU survivors and members of the public. Input from our patient partners relating to the lived knowledge of a patient journey following critical illness affirmed the need to have an individualized multicomponent approach to rehabilitation to address the complexities of the recovery process for survivors.

The construction of our intervention focused on *(d)* ‘ *identifying key uncertainties’* and *(e) ‘refining’* the intervention, and ( *f*) ‘ *economic considerations’* were made by considering the efficiency of using a core trained team to remotely deliver the intervention online.

Ethics approval was not required for this manuscript which reports intervention development and does not involve research data or participation.

### (i) Underpinning theory

***Components 1–4: goal setting and weekly action planning***

The iRehab intervention was informed by Leventhal’s Common-Sense Model (CSM) of self-regulation ^13, 14^. Leventhal’s CSM describes the process by which individuals respond to a perceived health threat, in this case their critical illness ^13^. It models how we think about our health by using common sense to interpret health threats and react to them in ways that may not always seem rational from the outside but have their internal logic. The model emphasizes several mechanisms that are important for adaptation to illness, including knowledge, past experiences, memories, and personal history. The model asserts that when a person is faced with a health problem, “an illness-related memory schema or ‘prototype’ is activated” ^13, 14^. Patients generally act to try to manage their condition or manage their ‘problem’; this is a self-regulation process, and it can evolve over time as illness progresses.

Patients use their perceptions of the illness (cognitive and emotional representations of the illness) to help them develop action and coping plans to manage the illness and find potential solutions (e.g., “I should call my doctor”; “maybe I’ll feel better if I rest”). Then, the ‘successes of these actions are appraised through constant feedback loop e.g.’ Am I less tired now’ ‘Am I better now?’) ^13^. Appraisal considers whether the strategy led to improvement, no change, or deterioration in symptoms, resulting in adjustment of the individual’s representation – this is a dynamic process. It has been proposed that clinicians can support and improve management/self-management by communicating with patients, clarifying their illness, supporting treatments, and helping with action plans ^15^. We incorporated these strategies into the intervention and included goal setting and weekly action planning ^16– 19^.

### (ii) Content based on post ICU outcomes, patient experience and evidence

***Component 1: Weekly discussion and individual symptoms management***

People experience common and varied symptoms after critical care, including dysphagia ^20^, breathlessness ^21^, fatigue ^22^, reduced appetite ^23^, delirium ^24^, and psychological symptoms including anxiety ^25^ and low mood/depression ^26^. Participants in our qualitative research identified the potential benefits of symptom management for recovery ^27, 28^. People wanted communication and guidance from healthcare professionals after critical illness “ *Encouragement from more people, within the health system*” *and “getting to know what your symptoms are*” ^29^. Views from our PAG about how helpful information sharing and weekly support from health professionals would be, informed the intervention. The iRehab intervention will address commonly occurring symptoms after critical illness, and care pathways will be prescribed based on individual needs ^29^. Evidence-informed treatment strategies have been embedded to help with individual symptom management. For example, breathing techniques for the management of breathlessness ^30^ and dietary advice for the management of poor appetite ^31, 32^.

***Component 2: Exercise and physical activity***

Physical impairment and deconditioning are common after critical care ^33^ and patients want advice about exercise and return to usual activities; they have indicated “there are certain physical things I would like to know about” ^27, 28^. Exercise and physical activity programs can aid physical and emotional recovery in people with chronic obstructive pulmonary disease, chronic fatigue, and congestive heart failure ^34– 37^. We previously tested an exercise intervention for people after critical care, which informed the exercise component for iRehab ^38^. We also drew on international experiences of remote multicomponent interventions e.g. pulmonary rehabilitation, liver surgery prehabilitation and cardiac rehabilitation; this informed the platform for remote delivery ^8, 39– 43^. Therefore, the inclusion of exercise and physical activity components was based on research and patient feedback.

***Component 3: Support for psychological wellbeing***

Patients often face a long journey from critical illness to recovery. Patients with lived experiences of critical illness told us about their challenges with their psychological wellbeing “ *I had terrible dreams when I was in ICU; nobody asked me once I came round about my ICU experience. And I have to say I was quite traumatised by it*,” and they wanted advice from someone who understood the sequelae after critical illness ^28^. Integrating an Acceptance and Commitment Therapy (ACT) based approach into a multidisciplinary intervention provides a clear framework for supporting individuals to set their own meaningful goals during that journey ^44^. The potential utility of the ACT approach is supported by previous research in related areas of physical health, demonstrating that ACT supports functioning and quality of life in life-limiting conditions, palliative care, chronic pain, and physical and mental health ^15, 44– 46^. Strategies for well-being activities, such as mindfulness or relaxation ^45, 47– 49^ as well as psycho-education related to delirium ^50^, are included in the iRehab program. The iRehab specialists delivering the intervention utilize resources based on ACT principles and committed action plans are reviewed with participants evolved on a weekly basis.

***Component 4: Group based peer support sessions and other information needs***

Participants in our qualitative research identified the potential benefit of speaking to others who have experience of recovery following discharge after an admission to ICU “maybe if I could speak to someone who had gone through the same experience as me” ^28^. In an international stakeholder engagement project involving patients, and families (n=66 interviews across the UK, Australia, and the US), the reported benefits of a peer-support intervention were 1) sharing experiences; 2) care debriefing (e.g., care understanding/navigation of health care system, external and internal validation of progress and feelings), and 3) altruism (e.g., a sense of purpose, giving back/helping others) ^51^. There is some evidence that peer support may reduce psychological morbidity in patients with critical illness ^52^. The inclusion of a peer support group in the iRehab intervention is based on this existing research, feedback from patients participating in our previous research on rehabilitation after critical illness, as well as input from our PAG members. Patients said that there was a “lack of information” about what happened in the ICU and what to expect during recovery, and they wanted this information to guide their recovery. Hence, information provision and peer support groups were included in the iRehab intervention.

### (iii) Constructing the intervention

The multiprofessional intervention development team (BO’N, JMB, BC, RC, ET, PF, SAD) used an iterative process (embedding the information above) to prepare, meet, obtain feedback, refine, and construct the intervention and its components. Input on a draft of the intervention was then sought from patient partners (n =11 current and past patient partners) and additional team members with relevant experience (DMcA, JB, JC). The content and construction of the iRehab intervention was also presented to an independent review panel of expert clinicians (n=5) (see acknowledgements) with critical care rehabilitation and intervention development expertise for feedback.

Materials were prepared for patients, and these underwent iterative review with specific input sought from the patient partners. The patient materials were assessed using the Drivel Defence Index to optimize the level of plain English ^53^. Our patient partners also provided feedback on video-based materials, such as exercise videos, recordings of treatment strategies, and the approach to providing these to participants. Intervention resources and a detailed intervention manual and training package were developed for use by trained iRehab intervention specialists to deliver key skills related to common problems managed after critical illness.

The intervention was developed to embed progressive intervention strategies capable of being delivered by a trained intervention team from a broad range of backgrounds. One iRehab specialist is assigned to one patient and provides their intervention across the 6 week programme. A multiprofessional group, each with specific expertise, will support and guide iRehab specialists in the management of patient symptoms and problems as required. iRehab specialists utilize resources which are defined as Level 1, where the content of the resource duplicates that of the patient materials, and Level 2, where the multiprofessional group member(s) provide additional specific guidance to the iRehab specialist to complement the use and implementation of the Level 1 resources. Level 2 could include an additional instruction, skill, or advice to iRehab specialists to facilitate their provision of individualized support to a participant, for example, instructions from the dietician about dietary modifications for a patient wanting to increase their intake of plant-based protein; advice from the physiotherapist about how to modify a specific exercise to change the muscle load or joint lever due to patient reporting tendinitis, neuropathy, or pain; or advice from the clinical psychologist to undertake a brief patient assessment, for example, completion of the generalized anxiety disorder questionnaire (GAD-7) to enable the psychologist to guide escalation of care. NICE (2011) recommends stepped models of care, which are of increasing relevance globally in response to high demand against a background of limited resources ^54^.

Key uncertainties were identified during intervention development, and refinements were made. For example, a key uncertainty is the use of remote delivery and our patient panel informed us about practical information to optimise remote delivery and communication with participants such as options to access computers or a phone, use of email and texts, opportunity for technical assistance from family or carers, and it was highlighted that specifically scheduling a weekly appointment slot is an important intervention element. It was also suggested that written intervention materials be provided to participants by post, with help available from the iRehab specialist to go through these printed materials with participants to optimize their understanding and application. Information for the intervention needed to be deliverable via phone/remotely; therefore, sections within the printed manuals were required to be color coded for easy identification of relevant sections during calls with participants.

## Results

### The iRehab intervention

The iRehab intervention is a patient centered, structured, individually tailored, multicomponent intervention designed to promote rehabilitation and recovery after critical illness. An overview of the rehabilitation intervention delivery format is shown in Table 1. It is intended to include a broad range of information, strategies, and support for people discharged home after mechanical ventilation in the ICU and at an early stage of the dynamic critical care recovery journey.

**Table 1.** Overview of iRehab rehabilitation intervention (Table 1 is sourced directly from https://doi.org/10.3310/nihropenres.13910.1).

Timing	Session format	Content
Week 1	1-to-1 appointment, online	Explain programme Assess symptoms and identify needs Provide individual symptom management Agree and start exercise and activity plan Agree review appointments
	Independent or online group/ recorded exercise session	Home exercise plan/attend group exercise session /access recorded exercise sessions
	Online peer support group (iRehab peer support cafe)	Attend iRehab peer support cafe
Weeks 2 to 6	1-to-1 appointment, online	Weekly review of symptoms and progression through treatment plans Continue needs assessment, symptom management and psychological support Complete live exercise and continue plan for weekly exercise/physical activity Agree review appointments
	Independent or online group/ recorded exercise session	Home exercise plan or attend group exercise session/access recorded exercise sessions
	Online peer support group (iRehab peer support cafe)	Attend iRehab peer support cafe
Week 6	Final 1-to-1 weekly session to include review and discharge	Encourage participant to continue with prescribed management plans Identify further sources of support Discharge from intervention.

The intervention is designed to allow for progression according to individual ability, building over six weeks, with weekly online, one-to-one appointments with a trained iRehab specialist, usually lasting up to one hour or more based on individual needs. Participants are required to continue with the agreed individual rehabilitation techniques at home and their progress should be reviewed, and action plans amended at weekly appointments. Participants are encouraged to attend a weekly group-based remote exercise session and a group-based remote peer support session (iRehab Peer Support Café).

The final iRehab intervention includes four core components (See Table 2 for further details).

**Table 2.** Details of the core components of the iRehab intervention.

Component	Aims	Content
**1. Weekly focused** **discussion and guidance** ** to determine** **individual symptoms ** **and management plans.**	To support rehabilitation and management of symptoms after critical illness through focused discussion and expert guidance to determine individual symptoms, and provide management strategies Objectives • Identify main symptom/issue from patient’s perspective • Provide strategies to address main symptoms and agree a goal/task for the week ahead using an Action Plan (AP)	• Participants identify symptoms of relevance to them in their recovery e.g. difficulty swallowing, breathlessness • Agree appropriate personalised management plan • Standardised treatment strategies, individualised to the patient’s needs will be provided. We have a menu of Level 1 * intervention strategies to address each symptom. • Option to consult with the relevant expert member of the multiprofessional team to provide a Level 2 * intervention; option to contact the site to identify an appropriate pathway if onwards referral is pending. • A goal/task for the week ahead will be discussed and agreed using an Action Plan (AP)
**2. Exercise and physical ** **activity**	To support rehabilitation and recovery and management of symptoms after critical illness through exercise and physical activity Objectives: • Deliver weekly exercise sessions and promote physical activity (walking) • Help the patient set goals and plan their exercise and physical activity for the week ahead and coping strategies to overcome barriers by using an Action Plan (AP)	• Participants will be supported to undertake progressive exercise and/or physical activity to manage physical weakness, deconditioning and reduced function and support wellbeing • Weekly live exercise session tailored to patient needs during the 1:1 session, plus plan to undertake at least one additional exercise session (either group based exercise, access a recorded session or use the exercise manual to guide their independent session) and/or increase physical activity (usually walking via time, distance, step counts). • Help the patient set goals and plan their exercise and PA for the week ahead using an Action Plan (AP) including coping strategies to help overcome barriers
**3. Support for** **psychological wellbeing**	To support rehabilitation and recovery through psychological and wellbeing approaches Objectives: • Conceptualise the individual’s situation utilising an Acceptance and Commitment Therapy focus using the ACT Matrix. • Identify appetitive (towards) and aversive (away) control • Define patients values and what gets in the way of connecting to a rich and meaningful life including cognitive fusion (internal) and experiential avoidance (external). • Develop a committed action plan and related psychoeducation on workable and unworkable strategies.	• Participants will be supported to manage any psychological symptoms e.g. anxiety, or low mood, delirium • Support for psychological wellbeing will specifically include an Acceptance and Commitment Therapy (ACT) focus. The iRehab specialists will be trained to use resources with interventions based on Acceptance and Commitment Therapy (ACT) principles and will have regular access (support and consultation) with an experienced clinical psychologist to provide further advice • Taught strategies or wellbeing activities e.g. mindfulness or relaxation will be incorporated into the patient action plan. • Strategies related to delirium include provision of the leaflet on delirium form ICU steps. • Action plans are reviewed weekly. If further input is needed and local services are available, the patient will be signposted onwards
**4. Group based peer ** **support sessions and** ** other information**	To support rehabilitation and recovery through peer support and addressing information needs Objectives: • Provide information about peer support and encourage attendance at the remote iRehab peer support group • Identify information needs and provide discussion, information, and sign posting to other relevant resources and services e.g. how and when to seek help and support, ICU steps, GP	• Participants will be encouraged to attend a weekly online iRehab peer support Café. A loose topic guide will be used to facilitate the session. • Based on individual needs and/or preference participants will also be provided with information about the critical care journey, how and when to seek help, and ongoing peer support.

*Level 1 is where the iRehab specialists’ resource or treatment strategy duplicates the patient materials; Level 2 is where the multiprofessional team member(s) provide additional specific guidance to compliment the use and delivery of the Level 1 resource such as an additional skill, instruction, or advice to facilitate the delivery or individualization of the skill/advice/support, for example, instruction from the dietician about diet modifications for a patient wanting to increase their intake of plant-based protein; or advice from the physiotherapist about how to modify a specific exercise to change the muscle load or joint lever due to pain, tendinitis, or neuropathy; or occasionally a suggestion from the clinical psychologist to undertake a brief assessment, for example, completion of the GAD to enable the psychologist to guide escalation of care.

1. Weekly discussion and guidance regarding symptom management;

2. Exercise and physical activity;

3. Support for psychological wellbeing;

4. Group based peer support and other information.

The intervention includes a range of strategies that can be adapted based on individual patient needs. The iRehab specialist can consult with the relevant expert member(s) of the multiprofessional team to assure their delivery of the ‘Level 1’ resources/intervention or identify an additional ‘Level 2’ intervention strategy. If there is a need for an onward referral, the intervention team can connect with the critical care team at the patient’s hospital site to identify an appropriate pathway or onwards referral or direct the patient to their GP. The intervention strategies provided to individual participants should be appraised at the weekly appointments through a constant feedback loop e.g. has the strategy helped or not, does the goal or treatment strategy need adjusted or the patient’s expectations; this is a dynamic process.

### iRehab Intervention team, training, materials, and mode of delivery

***Intervention team and training*:** The iRehab specialists will receive bespoke training, including access to the detailed intervention manual to support delivery, presentations, rehearsals, and interactive problem solving via case studies prior to delivering the intervention. They should be certified to deliver the iRehab intervention and supported with frequent and ongoing mentorship from an expert multiprofessional team, as well as training updates.

***Materials*:** An iRehab specialist’s detailed intervention manual contains instructions and information designed to enable them to support participants over a six-week period to set goals and plan their actions for exercise, physical activity, psychological well-being, and symptom management for the week ahead, along with any other activities in line with the components of the intervention. To minimize performance bias in intervention delivery, the core components in the manual are standardized and incorporate suggested scripts and protocols to guide overarching delivery, while still enabling flexibility in how these are applied to individual participants. The intervention is designed to be used alongside a range of participant materials, either printed copies or accessed via electronic versions for reference during remote rehabilitation sessions, after which supplemental information is provided according to patients’ feedback and individual needs.

***Access/Mode of delivery*:** The preferred mode of remote delivery (video or telephone) should be agreed with the participant. Guidance and help with troubleshooting the use of technology can be provided and/or an invite to have a relative or caregiver to assist.

## Discussion

A detailed description has been provided of the development (theory, content, and construction) of an intervention to support and promote rehabilitation and recovery after critical illness – the iRehab intervention. It was developed based on the core elements recommended by the MRC framework ^12^. The intervention is currently being tested in a RCT that will include rigorous process evaluation ( 10, iRehab Trial ISRCTN11266403). A logic model was developed to propose how the intervention could lead to its effects and under what conditions during the iRehab trial ( Table 3). If the intervention is effective the expectation is that improvements at 8 weeks will be observed in health-related quality of life per the EQ-5D-5L; also measures of leg strength/exercise capacity (30second sit to stand test), Illness Perception (Brief Illness Perception Questionnaire), Fatigue (FACIT-F), Hospital Anxiety and Depression Scale (HADS), patients satisfaction/acceptability (TFAQ)

**Table 3.** Logic model: to propose how the intervention could work within the iRehab trial, and relationships between resources, activities and outcomes.

INTERVENTION OBJECTIVE	INPUTS	OUTPUTS		OUTCOMES	IMPACT
	INTERVENTION RESOURCES	ACTIVITIES IN THE INTERVENTION	PARTICIPANTS	WHAT CHANGES DO WE EXPECT TO SEE	WHAT WILL BE DIFFERENT IF WE ARE SUCCESSFUL
To improve the health-related quality of life, physical function, fatigue, mood, and other health- related outcomes after critical illness	- iRehab specialist (trained and certified) and expert MDT - Ongoing mentorship - iRehab intervention manual - Patient manuals, trial documents and equipment (pulse oximeter) - Infrastructure & technology - Funding, Patient and public partners, Warwick Clinical Trials Unit, Research team, Hospital sites	Four key components - Symptom management - Exercise and physical activity - Psychological support - Peer support & information Delivery -Weekly 1:1 with iRehab specialist +/- -Weekly group exercise +/- -Weekly peer support group Taught skills and resources to address patient symptoms and needs. Weekly action plan for each patient.	Participants are people who have had an admission to the intensive care unit and are within 0–12 weeks of discharge home	Improvements at 8 weeks - Health-related quality of life – EQ-5D-5L - Physical Function – 30 second sit to stand test - Illness Perception – Brief Illness Perception Questionnaire - Fatigue – (FACIT-F) scale - Hospital Anxiety and Depression Scale (HADS) - Views of patients – satisfaction/acceptability via TFAQ - Views of others (Staff)	Participants will -perceive improved HR-QoL - be able to exercise and be stronger, fitter and more active -have increased self-management, self-regulation and confidence -feel satisfaction and improved wellbeing having received increased support and guidance from clinicians/others - feel their symptoms, mood and mental wellbeing will have improved There will be overall evidence that multicomponent rehabilitation delivered remotely is effective and cost effective

**Table:** 

**Moderating factors**	Physical performance at study outset Mental wellbeing at study outset	Perceived control and Self Efficacy	Credible source (iRehab specialist) Importance of feedback	Level of support available versus needed

**Table:** Assumptions

Patients will be interested and agree to take part and will participate in the intervention; staff will deliver the intervention as intended; sites will support recruitment of study participants; outcome measures will be collected as planned.

**Table:** External factors

Remote delivery in the participants home: there are advantages and disadvantages Not all participants will have IT for video call/likely at least 50% will take part via video call as planned and 50% by phone Intervention could become available locally across duration of study if service models change

Reference: How to develop a program logic for planning and evaluation Australian Institute of Family Studies https://aifs.gov.au/resources/practice-guides/how-develop-program-logic-planning-and-evaluation [last accessed 23.10.2024]

There is no agreed methodology for the development of interventions; several options exist, such as SQUID ^55^, Intervention Mapping ^56^, intervention taxonomy ^57^, the person-based approach ^58^, and the MRC framework ^12^. Key criteria often include understanding the problem, identifying what aspects could be changed and what change(s) would be helpful, identifying how to make changes by proposing a mechanism of change, designing and testing the intervention, and understanding the process of change through a process evaluation ^12, 55– 58^. However, a thorough description of the development process will help others to understand the level of complexity of the intervention and the rationale for its content.

We developed the iRehab intervention using evidence from existing literature, our previous research, views from patients with lived experience of critical illness, and clinical and research expertise from a broad multidisciplinary team and are confident that the intervention can be delivered as intended. Nonetheless, a lack of intervention adherence and low fidelity can influence trial outcomes ^59, 60^. To optimize the successful delivery of the iRehab intervention, the core components are fully protocolized to guide the delivery. In the iRehab trial, the intervention delivery team are trained to ensure that the delivery of core components is standardized. The trial includes a comprehensive process evaluation to fully assess all aspects of intervention fidelity and delivery. Active monitoring and early feedback will be implemented to prevent drift, ensure quality of delivery across staff and time, and monitor the inclusion of proscribed elements ^59, 60^. This manuscript describes intervention content in accordance with TIDieR guidance, and the results of the process evaluation will be published to ensure that all relevant insights into the intervention are available for interpretation ^61^. If the intervention is effective the trial manuals, resources and training package could be adapted for different healthcare systems beyond the UK NHS by considering for example, translations and cultural adaptations, and using a similar centralized staffing model for intervention delivery.

Since the development of this structured rehabilitation intervention, one randomized controlled trial testing rehabilitation in patients with critical illness has been published. Khan *et al.* 2024 found there was no improvement in QoL (SF36) in an intervention group that received a 12-month nurse-led collaborative care intervention (m-CCRP) supported by an interdisciplinary team compared to a control group ^62^. The trial population differs from the iRehab trial (e.g., shorter time with mechanical ventilation - 24 compared to >48 hours in iRehab), and while similarities include the use of protocols to support individual symptom management, there are several other differences, for example, there is no exercise component and contact (face to face initially) is infrequent and across 12 months. The iRehab remote intervention is designed to cover a broad range of possible strategies, delivered individually and in a group, with flexibility based on patient needs, and including goal setting and action plans, and delivered at levels one or two. The intervention is intended to be delivered to participants within 12 weeks of discharge from the hospital to facilitate positive readjustment once a patient has returned to life at home after critical illness. Testing our structured, individually tailored, multicomponent intervention will provide evidence whether rehabilitation delivered remotely is clinically and cost-effective for survivors following critical illness post ICU care.

The strengths of the iRehab intervention include its development by relevant key stakeholders and the inclusion of components that can be adapted and progressed according to individual ability and delivered remotely by a core trained intervention team. If effective, it has been designed to support efficient delivery when ICU staff resources are limited. It is delivered relatively early in a person’s post-hospital recovery journey. While theories and frameworks other than Leventhal’s CSM could have been used to inform this complex intervention, such as social learning theories ^63^, treatment and enablement theories ^64^, and the behavior change framework (COM-B) ^65^, it is difficult to make an optimal selection. However, in this intervention, the intention is to support participants with treatment strategies and the development of action and coping plans to help their rehabilitation and recovery, which is underpinned by Leventhal’s CSM. We anticipate that we will be able to successfully measure changes in illness perception and health outcomes using this approach ^66, 67^.

The theoretical model underpinning the psychological work with patients is based on ACT, an acceptance-based behavior therapy ^68^, and part of what is often known as the third wave of cognitive behavioral therapy (CBT). ACT therapy allows individuals to reconnect with their sense of self, identify meaningful values, and set goals in line with these values. The ACT Matrix will be utilized as a framework for guiding discussions with patients ^69^. Active engagement and goal setting require a reoriented physical and psychological sense of self; however, ICU experiences often result in a profound disruption to coping and identity, requiring guidance to support the development of purpose and meaning ^70, 71^.

Our intervention construction has limitations. First, we did not publish the specific methodology plan a priori for intervention development, although we did include key recommended criteria for intervention development, stakeholder involvement, and reviews ^12^. Second, other issues may need to be addressed, such as ways to support families of patients who have been in the ICU (although family members can be present to support participants if the patient wishes) and social prescribing (although participants are signposted to their GP for onward referrals) ^72, 73^.

## Conclusion

The consequences of critical illness are substantial and multifactorial. This paper reports the development of a rehabilitation program that is currently under evaluation within a rigorous clinical trial framework ^10^. The effectiveness and cost-effectiveness of the iRehab intervention compared to usual NHS care on quality of life and other health-related outcomes will be reported, and this intervention description will enable the interpretation of the trial findings.

## Ethics and consent

Ethics approval was not required for this manuscript which reports intervention development and does not involve research data or participation.

## Data Availability

No data are associated with this article
