# Supplementary material for: Development of a patient centred, structured, individually tailored, multi-component intervention to promote rehabilitation and recovery after critical illness: content, theory, and construction
Source: NIHR Open Res. 2025 Oct 6;5:64. Originally published 2025 Aug 4. [Version 2] doi: 10.3310/nihropenres.14023.2 (PMC12444276; doi:10.3310/nihropenres.14023.2)
Supplement: Peer review history

# Reviewer report: Reviewer response for version 2

**DOI:** 10.3310/nihropenres.15365.r37566

**Published:** 2025-10-20

**Authors:** Porkodi Arjunan

**Affiliations:** 1 King Faisal University, Al Hofuf, Saudi Arabia

1. **Participants – ICU population**
  Please clarify whether the study intends to include all post-ICU The authors described the participants are people who have had an admission to the intensive care unit and are within 0–12 weeks of discharge home. Justify the reason for taking all the patients admitted in ICU and also 12 weeks after discharge almost the patients recovered from the illness, then what is the purpose of implementing rehabilitation. The rehabilitation should start from the time of admission to the hospital.
2. **Exercise and physical activity- weekly live session**
  How is it possible to have live session one on one for exercise, in that case how many rehabilitations specialist will be needed to manage the exercise session. All ICU Patients may not require exercise/ rehabilitation. Justify the reason and feasibility 

Is the rationale for developing the new method (or application) clearly explained?

Yes

Is the description of the method technically sound?

Yes

Are the conclusions about the method and its performance adequately supported by the findings presented in the article?

Partly

If any results are presented, are all the source data underlying the results available to ensure full reproducibility?

No source data required

Are sufficient details provided to allow replication of the method development and its use by others?

Partly

Reviewer Expertise:

I have expertise in the area of heart failure and Cardiac rehabilitation, and ICU

I confirm that I have read this submission and believe that I have an appropriate level of expertise to confirm that it is of an acceptable scientific standard, however I have significant reservations, as outlined above.

# Reviewer report: Reviewer response for version 2

**DOI:** 10.3310/nihropenres.15365.r37565

**Published:** 2025-10-10

**Authors:** Andreas Xyrichis

**Affiliations:** 1 King's College London, London, England, UK

Thank you. I have no further comments.

Is the rationale for developing the new method (or application) clearly explained?

Yes

Is the description of the method technically sound?

Yes

Are the conclusions about the method and its performance adequately supported by the findings presented in the article?

Yes

If any results are presented, are all the source data underlying the results available to ensure full reproducibility?

No source data required

Are sufficient details provided to allow replication of the method development and its use by others?

Yes

Reviewer Expertise:

Health services research

I confirm that I have read this submission and believe that I have an appropriate level of expertise to confirm that it is of an acceptable scientific standard.

# Reviewer report: Reviewer response for version 1

**DOI:** 10.3310/nihropenres.15251.r36827

**Published:** 2025-09-17

**Authors:** Andreas Xyrichis

**Affiliations:** 1 King's College London, London, England, UK

This manuscript presents a comprehensive account of developing the iRehab intervention for post-critical illness rehabilitation. The work addresses a significant clinical need and demonstrates rigorous adherence to established frameworks for complex intervention development.

 There is much to commend about this article:

 1. Methodological rigour and framework adherence: The authors demonstrate commendable adherence to the MRC framework for complex intervention development, systematically addressing each core element (a-f). The integration of multiple evidence sources—existing literature, psychological theory, patient experiences, and clinical expertise—reflects thorough intervention development methodology. The use of TIDieR reporting guidelines enhances transparency and supports replication efforts.

 2. Theoretical foundation and integration: The selection of Leventhal's Common-Sense Model provides a coherent theoretical foundation for supporting patient self-regulation following critical illness. The integration with Acceptance and Commitment Therapy principles for psychological wellbeing creates a complementary framework that addresses both cognitive and behavioural aspects of recovery. This dual theoretical approach appropriately reflects the complexity of post-ICU rehabilitation needs.

 3. Patient-centred development approach: The extensive patient and public involvement represents a strength of this work. The formation of a Patient Advisory Group with ongoing involvement throughout development, trial conduct, and dissemination planning demonstrates genuine co-production rather than tokenistic consultation. The patient partners' contribution to the plain language summary exemplifies meaningful collaboration and enhances accessibility.

 4. Intervention design and flexibility: The four-component structure (symptom management, exercise/physical activity, psychological support, peer support) addresses the multifaceted nature of post-critical illness syndrome comprehensively. The two-level resource system (Level 1 standardised, Level 2 specialist-guided) strikes an appropriate balance between standardisation for research purposes and individualisation for clinical effectiveness. This graduated approach reflects real-world clinical decision-making processes.

 5. Remote delivery innovation: The adaptation for remote delivery demonstrates forward-thinking design, particularly relevant given recent healthcare delivery changes. The flexibility to deliver via video or telephone, supported by posted materials, addresses potential technology barriers while maintaining intervention accessibility. The consideration of practical implementation barriers (technology access, family support) shows thoughtful intervention design.

 6. Logic model and outcome alignment: Table 3 provides a clear logic model linking intervention inputs through to anticipated impacts. The alignment between intervention components and proposed outcome measures (EQ-5D-5L, physical function tests, illness perception measures) demonstrates coherent intervention theory. The inclusion of moderating factors and external considerations shows sophisticated understanding of implementation contexts.

 7. Quality assurance and fidelity planning: The detailed training requirements for iRehab specialists, standardised manual development, and planned process evaluation demonstrate commitment to intervention fidelity. The multiprofessional expert review and iterative refinement process enhances intervention credibility and implementation readiness.

 Minor Suggestions

 1. Consider briefly discussing how the intervention might be adapted for different healthcare systems beyond the UK NHS.

 2. The logic model could benefit from explicit mention of cost-effectiveness pathways given the economic evaluation component.

Is the rationale for developing the new method (or application) clearly explained?

Yes

Is the description of the method technically sound?

Yes

Are the conclusions about the method and its performance adequately supported by the findings presented in the article?

Yes

If any results are presented, are all the source data underlying the results available to ensure full reproducibility?

No source data required

Are sufficient details provided to allow replication of the method development and its use by others?

Yes

Reviewer Expertise:

Health services research

I confirm that I have read this submission and believe that I have an appropriate level of expertise to confirm that it is of an acceptable scientific standard.

# Response

**Published:** 2025-09-26

**Authors:** Brenda O'Neill

**Affiliations:** Ulster University Centre for Health and Rehabilitation Technologies, Belfast, Northern Ireland, UK

**Thank you for your review of our manuscript. We really appreciate your helpful comments and suggested edits**

 1. Consider briefly discussing how the intervention might be adapted for different healthcare systems beyond the UK NHS

**Response- We have considered this and added a suggestion to the discussion.**

**Please see discussion section, page 11, after paragraph 2**

**“If the intervention is effective the trial manuals, resources and training package could be adapted for different healthcare systems beyond the UK NHS by considering for example, translations and cultural adaptations, and using a similar centralised staffing model for intervention delivery.”**

 2. The logic model could benefit from explicit mention of cost-effectiveness pathways given the economic evaluation component.

**Response- Thank you: we could consider this although it would be challenging as it would bring much additional detail to the current logic model.** **The trial plans a cost analysis that will include economic modelling. We will suggest to the trial Health Economists on the team to consider the development of a separate logic model to more fully propose the relationships and interactions between the intervention costs and trial outcomes.**

# Reviewer report: Reviewer response for version 1

**DOI:** 10.3310/nihropenres.15251.r36828

**Published:** 2025-09-05

**Authors:** David M Griffith

**Affiliations:** 1 University of Edinburgh, Edinburgh, UK

Thank you for the opportunity to review this excellent paper which describes the process of development of a the iRehab intervention.  The manuscript is well written, and the reporting and development of the intervention follows recognised guidance.  What is particularly impressive is the active participation of patients and members of the public in the development of the intervention, the ongoing conduct of the trial, and in the drafting and reporting of this manuscript.  The resulting intervention is indeed patient-centred I would expect it to be well received by patients as a result. 

 My comments relate to the alignment of the trial intervention with the trial outcome measures, given this has been a limitation of many previous post-ICU studies.  It is not clear whether the trial outcome measures were considered during the development phase and there is no reflection in the discussion about how well the outcome measures will capture the success of the intervention. 

 Specifically:

1. The manuscript does not specifically mention the primary (or secondary) outcome measures used in iRehab (EQ-5DL) could the authors include this information in the manuscript?
2. How were the trial outcomes considered in the development of the intervention?
3. Could the authors reflect on the alignment between the intervention and the iRehab primary outcome measure. 
4. Pain is a common issue in ICU survivors.  Given that pain/discomfort is one of the 5 dimensions of EQ-5D-5L, could the authors mention specifically how the intervention will address pain issues in the post-ICU period.

Is the rationale for developing the new method (or application) clearly explained?

Yes

Is the description of the method technically sound?

Yes

Are the conclusions about the method and its performance adequately supported by the findings presented in the article?

Yes

If any results are presented, are all the source data underlying the results available to ensure full reproducibility?

No source data required

Are sufficient details provided to allow replication of the method development and its use by others?

Yes

Reviewer Expertise:

Critical care recovery.  Critical Care.  Post-ICU weakness.

I confirm that I have read this submission and believe that I have an appropriate level of expertise to confirm that it is of an acceptable scientific standard, however I have significant reservations, as outlined above.

# Response

**Published:** 2025-09-26

**Authors:** Brenda O'Neill

**Affiliations:** Ulster University Centre for Health and Rehabilitation Technologies, Belfast, Northern Ireland, UK

**Thank you for reviewing our manuscript and the helpful comments and edits which have improved our reporting. Please find our responses to your comments below.**

1. The manuscript does not specifically mention the primary (or secondary) outcome measures used in iRehab (EQ-5DL) could the authors include this information in the manuscript?

**Response - The logic model presented in Table 3 proposes how the intervention within the iRehab trial could change the trial outcome measures - see column ‘What changes do we expect to see’.**

**We refer to the logic model in the discussion section but for better visibility we have added the following amendment**

**See discussion, Page 10, paragraph 1**

**“ *A logic model was developed to propose how the intervention could lead to its effects and under what conditions during the iRehab trial (Table 3).”***

**amended to**

***“A logic model was developed to propose how the intervention could lead to its effects and under what conditions during the iRehab trial (Table 3). If the intervention is effective the expectation is that improvements at 8 weeks will be observed in health-related quality of life per the EQ-5D-5L; also measures of leg strength/exercise capacity (30second sit to stand test), illness perception (Brief Illness Perception Questionnaire), fatigue (FACIT-F),  anxiety and depression (HADS), patient  satisfaction/acceptability (TFAQ).***

 2. How were the trial outcomes considered in the development of the intervention?

**Response - The trial outcomes were considered in the context of the proposed causal pathway of the intervention, literature identifying post ICU sequalae, and relevant core outcome sets (Table 3 and references 44, 45, 48).**

**The objective of the intervention is “To improve the health-related quality of life, physical function, fatigue, mood, and other health related outcomes after critical illness.” As the intervention is multi-component, underpinned by theory and research, and tailored to patients’ needs it is anticipated it will contribute to several dimensions that underpin health-related quality of life.**

**The core outcome set for long-term outcomes following** **acute respiratory failure (ref 44)** **indicates EQ-5D-5L is the preferred option for measuring health-related quality of life. with SF36 a secondary option. Recent reviews (ref 46, 47) have concluded the EQ-5D-5L is as good as other health related QOL outcome measures (e.g. SF36), and responsive to multicomponent rehabilitation in patients after critical illness (48).**

**Furthermore, recognising that HRQoL has multiple components and means different things to different people it was important to include secondary outcomes (such as leg strength/endurance, fatigue, mood) that link to common problems identified after critical illness.**

 3. Could the authors reflect on the alignment between the intervention and the iRehab primary outcome measure.

**Response - For the iRehab trial, the primary outcome is health-related quality of life, measured using the EQ-5D-5L at eight weeks post-randomisation. Health-related quality of life is multidimensional, encompassing components of physical, psychological, emotional, and social wellbeing. The EQ-5D-5L is the recommended measure for assessing quality of life in core outcome sets for longer-term outcomes following respiratory failure and physical rehabilitation in critical illness (44,45).** **S** **ystematic reviews confirm the EQ-5D-5L to be similarly robust compared with other longer measures, such as the SF-36 (46,47). The scale** **measures the extent of impairment in** **mobility, self-care, usual activities, pain/discomfort, and anxiety/depression, ranging from no problems to severe problems. T** **hese domains, and the** **visual analogue scale (VAS 0-100mm) capturing health utility, are** **widely used for health economic evaluation. The scale was responsive to change in a study assessing multicomponent rehabilitation in post-critical illness patients (48), and has been validated for telephone completion. Importantly, our patient and public partners endorsed quality of life as an important outcome to reflect recovery after critical illness.**

**Additional advantages of the EQ-5D-5L are that it is concise, short, suitable for remote completion, and supports a health economic evaluation.**

 4. Pain is a common issue in ICU survivors. Given that pain/discomfort is one of the 5dimensions of EQ-5D-5L, could the authors mention specifically how the intervention will address pain issues in the post-ICU period.

**Response - The intervention includes the opportunity for participants to discuss any problems/symptoms they have since going home from hospital with their iRehab specialists; a range of resources are available to help. If pain is identified as a problem/symptom this could be addressed by the patient receiving advice and strategies from a specific leaflet about “pain after critical illness;” and they could be signposted to see their general practitioner if they need pharmacological input or an alternative onward referral.**

**Because there is some overlap between pain and other symptoms e.g. sleep (poor sleep due to pain), mental health (higher perception of pain when anxious) and higher pain levels due to muscle weakness/reduced function, the patient might also receive strategies targeted to improving these e.g. acceptance and commitment therapy and mindfulness, tips for better sleep, modifications to enable a suitable amount of exercise; and adaption of weekly goals to be more achievable within pain boundaries.**

**To help link the dimension of pain to the intervention we have included pain as an example symptom in Table 2, Column 3 “Content”**

**Line 1 **

- **“Participants identify symptoms of relevance to them in their recovery e.g. difficulty swallowing, breathlessness**

**amended to**

- **“Participants identify symptoms of relevance to them in their recovery e.g. difficulty swallowing, breathlessness, pain**

# Reviewer report: Reviewer response for version 1

**DOI:** 10.3310/nihropenres.15251.r36677

**Published:** 2025-09-05

**Authors:** Porkodi Arjunan

**Affiliations:** 1 King Faisal University, Al Hofuf, Saudi Arabia

This manuscript addresses an important and timely topic in post–intensive care rehabilitation. The study has potential significance; however, the current version requires substantial clarification of participant selection, the definition and scope of the iRehab specialist, standardization of the intervention across settings, and patient safety procedures.

 Specific Comments

1. **Participants – ICU population**
  Please clarify whether the study intends to include all post-ICU patients across subspecialties (e.g., cardiothoracic, neuro, transplant, burns) or focus on specific ICU populations. Given the heterogeneity in recovery needs, justification is required for the chosen population.
2. **Definition of the iRehab specialist**
  The role of the “iRehab specialist” is not clearly defined. It is unclear whether this refers to a single individual or a multiprofessional team. Please clarify the team composition, professional background, and responsibilities. Additionally, explain whether the iRehab specialist will strictly follow the primary physician’s plan or develop an independent rehabilitation plan, and how multiprofessional input will be integrated.
3. **Duration of post-ICU management**
  The proposal to provide six weeks of uniform post-ICU management for all patients needs justification. Not all post-ICU patients may require this duration of follow-up. Please explain the rationale for a uniform 6-week intervention and how variation in patient needs will be addressed.
4. **Exercise intervention feasibility**
  The manuscript should clarify how the program will apply to patients unable to participate actively in exercise. In cases where patients require only passive movements, explain how the intervention remains applicable and beneficial
5. **Safety considerations during exercise**
  Please describe how complications or adverse events arising during exercise sessions will be identified and managed. A safety monitoring plan is essential for patient protection.
6. **Participant timeframe (0–12 weeks post-discharge)**
  The recruitment timeframe of 0–12 weeks post-discharge appears broad. By 12 weeks, many patients may already have regained near-normal function. Please justify this wide window and consider narrowing the inclusion timeframe. 
7. **Intervention standardization**
  As hospital policies and discharge practices differ, it is important to clarify how the intervention will be standardized across centers. Please describe how consistency will be ensured in delivering the iRehab program despite institutional differences.

Is the rationale for developing the new method (or application) clearly explained?

Yes

Is the description of the method technically sound?

Yes

Are the conclusions about the method and its performance adequately supported by the findings presented in the article?

Partly

If any results are presented, are all the source data underlying the results available to ensure full reproducibility?

No source data required

Are sufficient details provided to allow replication of the method development and its use by others?

Partly

Reviewer Expertise:

I have expertise in the area of heart failure and Cardiac rehabilitation, and ICU

I confirm that I have read this submission and believe that I have an appropriate level of expertise to confirm that it is of an acceptable scientific standard, however I have significant reservations, as outlined above.

# Response

**Published:** 2025-09-26

**Authors:** Brenda O'Neill

**Affiliations:** Ulster University Centre for Health and Rehabilitation Technologies, Belfast, Northern Ireland, UK

**Thank you for your detailed review and the helpful comments and suggestions which have improved our reporting. Please find our response to your comments below.**

1. **Participants – ICU population**

Please clarify whether the study intends to include all post-ICU patients across

 subspecialties (e.g., cardiothoracic, neuro, transplant, burns) or focus on specific ICU

 populations. Given the heterogeneity in recovery needs, justification is required for the

 chosen population.

**Response - **The study intends to include people who received invasive mechanical ventilation for 48 hours or longer during an ICU admission. The inclusion criteria were selected based on evidence for poor recovery in these patients.. Full details of the inclusion and exclusion are available in the study protocol [ref 10, https://openresearch.nihr.ac.uk/articles/5-29 ]

 All eligible patients will be included in the trial, regardless of subspecialty unless an established rehabilitation pathway exists for their management in local practice.  In this case, patients would not be included and would access that particular care pathway.

 The reference to the paper detailing the iRehab trial protocol has now additionally been included in the manuscript conclusion so that readers can access the iRehab trial inclusion criteria and additional deails.

 see Conclusion/page 11

 “This paper reports the development of a rehabilitation program that is currently under evaluation within a rigorous clinical trial framework (10)……..”

**2. Definition of the iRehab specialist**

 The role of the “iRehab specialist” is not clearly defined. It is unclear whether this refers to a single individual or a multiprofessional team. Please clarify the team composition, professional background, and responsibilities. Additionally, explain whether the iRehab specialist will strictly follow the primary physician’s plan or develop an independent rehabilitation plan, and how multiprofessional input will be integrated.

**Response - **One iRehab specialist is assigned to one patient to provide their intervention across the whole 6 week programme The team of trained iRehab specialists deliver the group sessions in pairs.

 Rather than a physician-planned intervention the iRehab specialists will develop a patientcentred rehabilitation plan following the instructions from the iRehab intervention manual and training .

 The text has been edited to clarify this

 see p 7 “(iii) Constructing the intervention

 The intervention was developed to embed progressive intervention strategies capable of being delivered by a trained intervention team from a broad range of backgrounds. A multiprofessional group, each with specific expertise, is expected to support and guide iRehab specialists in the management of patient symptoms and problems as required. iRehab specialists utilize resources ……..”

 amended to

 see p 7 “(iii) Constructing the intervention

 The intervention was developed to embed progressive intervention strategies capable of being delivered by a trained intervention team from a broad range of backgrounds. “One iRehab specialist is assigned to one patient and provides their intervention across the 6 week programme” A multiprofessional group, each with specific expertise, will support and guide iRehab specialists in the management of patient symptoms and problems as required. iRehab specialists utilize resources ……..”

 For information the iRehab intervention specialists are a team of trained professionals with experience of working with critical care patients and/or delivering rehabilitation to clinical populations. Key criteria for these roles include a graduate qualification in a relevant subject e.g. Physiotherapy, Occupational Therapy, Sport and Exercise Science, Nursing. iRehab intervention specialists are supported by regular meetings with a multiprofessional team to review intervention delivery and address any specific participant queries.  This multiprofessional team has extensive expertise in the management of patients after critical illness.

**3. Duration of post-ICU management**

 The proposal to provide six weeks of uniform post-ICU management for all patients needs justification. Not all post-ICU patients may require this duration of follow-up. Please explain the rationale for a uniform 6-week intervention and how variation in patient needs will be addressed.

**Response- **The 6 week approach was based on literature and programmes in other populations such as cardiac and pulmonary rehabilitation, as well as data from our preliminary exercise-based study in this population (references 26, 37).

 Variation in patient needs are addressed through the range of components that make up the intervention outlined in Table 2.

 The duration of the programme that best suits patients is important. Therefore, we will gather data on whether participants complete all sessions (adherence) and this will give insight into whether patients discontinue the intervention before the 6 week period or not (reporting per TIDIER in the results manuscript).

**4. Exercise intervention feasibility**

 The manuscript should clarify how the program will apply to patients unable to participate actively in exercise. In cases where patients require only passive movements, explain how the intervention remains applicable and beneficial

**Response-  **It is extremely unlikely that a patient who meets the study criteria and is discharged home from hospital would require only passive movements given the trial inclusion /exclusion criteria. The inclusion criteria include being discharged from hospital and able to participate in the intervention and with trial procedures e.g. using equipment such as computer or telephone, and exclusion criteria include having no ‘contra-indications to exercise’ and not being ‘discharged to a care home with/without nursing care’.

**5. Safety considerations during exercise**

 Please describe how complications or adverse events arising during exercise sessions will be identified and managed. A safety monitoring plan is essential for patient protection.

**Response- **Exclusion criteria for trial recruitment include having no “Contra-indication to exercise” and these are based on the American College of Sports Medicine [2017]. Training specific to this criterion is provided to recruiting staff during the Site Initiation Visit (SIV) and iRehab specialists and responses to common queries are included in their trial manuals and a Frequently Asked Questions (FAQ) document on the trial website. Staff are also advised and encouraged to discuss any concerns or queries about contra-indication to exercise with the PI and the iRehab study team.

 For each patient, readiness and risk to exercise is explored on an individual basis by considering information from the recruitment site and trial database about for example, patients’ diagnosis and comorbidities, as well as the intervention manual which the trained iRehab specialist follows to ensure safety. For example, the iRehab specialist will assess resting oxygen saturation, or the need for a family member to be nearby, ability to undertake lower intensity exercise versus higher intensity exercise.

 During the iRehab trial, Adverse Events (AEs) and Serious Adverse Events SAEs will be assessed and reported in keeping with regulatory requirements. All staff follow the trial protocol and training to identify and manage AEs or SAEs. We have defined escalation plans for participants who experience a fall or are at risk of mental health crisis. The iRehab specialists delivering the intervention are also trained to optimise safety for undertaking exercise.

**6. Participant timeframe (0–12 weeks post-discharge)**

 The recruitment timeframe of 0–12 weeks post-discharge appears broad. By 12 weeks,

 many patients may already have regained near-normal function. Please justify this wide

 window and consider narrowing the inclusion timeframe.

**Response- **This is an important point. Research shows that people experience variable symptoms after discharge home following ICU and recovery trajectories are unpredictable with morbidity reported even years after ICU discharge (described in the iRehab trial protocol paragraph 1 and references 2-11 https://openresearch.nihr.ac.uk/articles/5-29 ). The optimal timing of intervention delivery is unknown (early versus later in the recovery trajectory) and we wanted to provide an opportunity for delivery of rehabilitation in the early discharge phase as this has been identified by patients as a period of high adjustment and when patients have multiple physical and mental well-being problems present (de silve et al 2024, Ferguson et al 2019, Iwashyna et al 2012); our patient partners also agreed this timing and we had previously shown it was feasible to recruit and deliver rehabilitation during this phase (McDowell et al 2017).

 During the trial we will gather data about the number of weeks between participants being discharged from hospital and when they are recruited and this will give insight into whether a wide or narrow window is optimal (we will report this in the trial results manuscript).

**7. Intervention standardization**

 As hospital policies and discharge practices differ, it is important to clarify how the

 intervention will be standardized across centers. Please describe how consistency will be ensured in delivering the iRehab program despite institutional differences.

**Response- **The trial protocol (reference 10) clarifies this: standard NHS care is the comparator group. The standard care and post ICU pathways at each site are identified to enable the team to ensure separation of trial arms. Sites are also actively monitored during the trail to gauge any changes that may necessitate reconsidering site eligibility. Sites delivering a similar intervention to the iRehab intervention are excluded.

 During the trial the iRehab intervention is delivered only by a centralised team of trained and certified iRehab specialists, that are independent of the recruitment sites/centres. To minimise performance bias in intervention delivery, the core components are protocolised to guide overarching delivery, whilst still enabling flexibility in how components are applied to individual participants. Active monitoring and early feedback by the independent process evaluation team will be implemented to ensure intervention fidelity; this is identified in the trial protocol [reference 10]
